# Supplementary material for: JAK and MEK Pathway Regulation of Mitochondrial Activity as Possible Targets for Saphenous Vein Smooth Muscle Cell Dysfunction in Diabetes
Source: Cells. 2026 Aug 3;15(15):1402. doi: 10.3390/cells15151402 (PMC13465089; doi:10.3390/cells15151402)
Supplement: Supplementary file 1 [file cells-15-01402-s001.zip › cells-4389845-supplementary.pdf]

SUPPLEMENTARY INFORMATION

Table S1: Materials

**Company/Supplier**	**Catalogue number**
**Abcam, Cambridge, UK**	
Ruxolitinib	ab141356
Oligomycin	ab141829
Carbonyl cyanide p-(trifluoromethoxy) phenylhydrazone (FCCP)	ab120081
Rotenone	ab143145
Antimycin A	ab141904
Anti-STAT3 antibody (EPR787Y)	ab68153
**Agilent Technologies Incorporated, Santa Clara, USA**	
Seahorse XF FluxPaks	103022-100
XF DMEM Medium, pH 7.4, 500ml, with 5 mm HEPES, without phenol red, sodium bicarbonate, glucose, sodium pyruvate, L-glutamine	103575-100
**Cell Signalling Technology, Massachusetts, USA**	
Phospho-Stat3 (Tyr705) antibody	9131
Phospho-p44/42 MAPK (Erk1/2) (Thr202/Tyr204) (E10) mouse antibody	9106L
p44/42 MAPK (Erk1/2) antibody	9102L
**PromoCell GmbH, Heidelberg, Germany**	
Smooth muscle cell growth medium 2 kit	C-22162
**R&D systems, Minnesota, USA**	
Recombinant human IL-6 protein	206-IL
Recombinant human IL-6 R alpha protein	227-SR-025
**Sigma-Aldrich (Merck), Dorset, UK**	
Ammonium persulfate (APS)	A3678
Hepes	H3375
Fetal bovine serum	F7524
Angiotensin II human	A9525-5mg

Thrombin from bovine plasma	T4648-1ku
**Stratech, Cambridgeshire, UK**	
Trametinib	GSK1120212
**Thermo Scientific, Massachusetts, USA**	
Recombinant human PDGF-BB	10531285

Table S2: Patients' characteristics with medication history and sample ID number

Patient ID	Age	Sex	Ethnic origin	Reason for surgery	Medical History	T2DM	Medication history
**TP-HSV001**	69	Male	British White	Coronary artery disease	T2DM, hypertension, hypercholesterolaemia	Yes	Metformin, atorvastatin, bisoprolol
**TP-HSV002**	70	Male	British White	Coronary artery disease	Hypertension, hypercholesterolaemia.	No	Aspirin, metformin, ramipril, ISMN, rosuvastatin, omeprazole, dapagliflozin
**TP-HSV003**	84	Male	British White	Coronary artery disease	Hypertension, hypercholesterolaemia	No	Aspirin, amlodipine, atorvastatin, bisoprolol
**TP-HSV004**	73	Male	British White	Coronary artery disease, aoritc valve stenosis	Heart attack, peripheral vascular disease, rectal cancer	No	Allopurinol, clopidogrel, latanoprost, ramipril
**TP-HSV005**	58	Male	British	Coronary artery disease, recent MI: Jan 2020	Heart attack, hypertensive, asthmatic	No	Aspirin, ticagrelor, isosorbibe dinitrate, bsoprolol, ramipril, artovastatin, spiromax.
**TP-HSV006**	77	Male	British	Coronary artery disease	T2DM, MI (2000), Basal cell carcinoma (removed), IHD, epilepsy	Yes	Carbamazipine, verapamil, rosuvastatin, clopidogrel
**TP-HSV007**	66	Male	British	Coronary artery disease	MI (2000), hypothyroidism, myeloid dysplasia disorder	No	Amlodipine, nicorandil, perindopril

**TP-HSV008**	61	Male	White	Triple vessel disease	High cholesterol	No	Aspirin, bisoprolol, isosorbide mononitrate, atorvastatin, amlodipine, sertraline
**TP-HSV009**	69	Male	Caucasian	Coronary artery disease	Heart attacks (2003), BPH, Cholesterol +HTL	No	Aspirin, bisoprolol, colecalciferol, eplerenone, ESMN, ramipril atorvastatin, ranitidine, tamsulosin
**TP-HSV010**	68	Male	White British	Coronary artery disease	T2DM, hypertension, hypercholesterolaemia	Yes	Metformin, atorvastatin, bisoprolol
**TP-HSV011**	69	Male	White	Coronary artery disease	T2DM, heart attack, hypercholesterolaemia	Yes	Bisoprolol, atorvastatin, amlodipine, omeprazole, metformin and aspirin
**TP-HSV012**	73	Male	White	Coronary artery disease	High cholesterol	No	Isorsorbide(stopped due to surgery), bisoprolol, aspirin, allopurinol, tamsulosin, simvastatin and ramipril
**TP-HSV013**	48	Male	White	Coronary artery disease	Heart attack (August 2020), Kidney failure, Asthma, hypercholesterolaemia and recent sepsis	No	Carvedilol, furosemide, salbutamol, doxazosin, atovastatin, lanzoprazole and ISMN
**TP-HSV014**	69	Male	White	Worsening angina	Not Known		Isorsorbide mononitrate, bisoprolol, aspirin, atovastatin and ramipril

**TP-HSV015**	54	Male	White	Coronary artery disease	Heart attack	No	Ramipril,atorvastatin, lansoprazole, aspirin, apixaban and lercanidipine
**TP-HSV016**	80	Male	White	Coronary artery disease	T2DM, Prostate CA, HTN, Carotid Artyery disease	Yes	Clopidogrel, bisoprolol, amlodipine, ISMN atorvastatin, Lansoprazole, Ranolazine, tamsulosin
**TP-HSV017**	73	Male	White British	Coronary artery disease	Hypertension	No	Isorsorbide mononitrate, atenolol, amlodipine, atorvastatin, clopidogrel and nitrolingual pump spray
**TP-HSV018**	63	Male	White British	Coronary artery disease	T2DM, hypercholesterolaemia	Yes	Empagliflozin, Humilin mix, aspirin, losartan, amlodipine, atorvastatin and Sukkarto SR
**TP-HSV019**	63	Male	White British	Double Coronary vessel disease	Heart attack, Carpal tunnel syndrome, Osteoathritis, Hypertension, Varicose vein	No	Empagliflozin, Carvedilol, Omeprazole, Glyceryl trinitrate, Cocodamol, Lactulose, Aspirin, Ramipril, Ezetimibe, Tildem Retard, Furosemide and Fenbid Firte (10% Gel)
**TP-HSV020**	68	Male	White	Coronary artery disease	Heart attack, left knee replacement, Osteoathritis	No	Amlodipine, Gabapentin,Aspirin, Bisoprolol,Atorvastatin and Omeprazole

**TP-HSV021**	74	Male	White	Coronary artery disease	Heart attack, Perivascular vascular disease,Rheumathoid and Osteo-athritis	No	Amlodipine, Lanzoprazole,Citalopram, Paracetamol, Aspirin, Loperamide, Ramipril, Atorvastatin and Isorsobide mononitrate
**TP-HSV022**	68	Male	White British	Coronary artery disease	Hypertension, Hypercholesteroleamia	No	Aspirin, Bisoprolol,Atorvastatin and Omeprazole
**TP-HSV023**	72	Male	White	Coronary artery disease	T2DM, Gout, GORD, Hypertension, Hypercholesteroleamia and BPH	Yes	Amlodipine, Lansoprazole,Clopidrogel, Doxazosin, Spironolactone, Atorvastatin, Finasteride, Allopurinol and Lercanidipine
**TP-HSV024**	63	Male	White British	Coronary artery disease	T2DM and heart attack	Yes	Aspirin, Bisoprolol,Atorvastatin, Lanzoprazole and Ramipril.
**TP-HSV025**	73	Male	White British	Coronary artery disease	Heart attack and hypertension	No	Ticagrelor, Amlodipine, Lanzoprazole,Deltapapin, Pantoprazole, Aspirin, Ramipril, Atorvastatin and Bisoprolol.
**TP-HSV026**	70	Male	White	Triple vessel disease	Covid 19 +ve, now -ve	No	Aspirin, Bisoprolol, Bimatropost and Ramipril

**TP-HSV027**	76	Male	White British	Coronary artery disease- recent myocardial infarction	Heart attack, Hypercholesterolemia and Gout	No	Throxin, atorvastatin, calcisine and lansoprazol
**TP-HSV028**	52	Male	White	Coronary artery disease	Heart attack and Hypercholesterolemia	No	Isosorbide mononitrate. Aspirin, Ramipril and Atorvastatin
**TP-HSV029**	75	Male	White British	Coronary artery disease	T2DM, Hypertension, Parkinson and Dyslipidaemia	Yes	Lansoprazole, metformin, aspirin, bisoprolol, co-caveldopa, furosemid, gliclizide, irbesatan, simvastatin and sitagliptin.
**TP-HSV030**	59	Male	White British	Coronary artery disease	Heart attack (2018)	No	Omeprazole, Losartan, Paracetamol, Aspirin, Oxycodone, Atorvastatin, isorsorbide mononitrate, and Bisoprolol.
**TP-HSV031**	72	Male	White British	Coronary artery disease	Sleep apnea, Gout, Diverticulosis, Bilateral osteoarthritis, shoulder replacement.	No	Allopurinol, Bisoprolol, Amiodarone, Furosemide, Lanzoprazole, Atorvastatin, isosorbide mononitrate, and Malirogol.
**TP-HSV032**	55	Male	White British	Coronary artery disease	Heart attack	No	Bisoprolol, Ramipril, Aspirin,Ticagrelor, Atovastartin, isosorbide mononitrate and Vitamin D3

**TP-HSV033**	57	Male	White British	Coronary artery disease	Heart attack, Hypertension and Hypercholesteroleamia	No	Aspirin, Amlodipine,Atorvastatin, Ramipril and Bisoprolol
**TP-HSV034**	82	Male	White British	Coronary artery disease	Cancer	No	Aspirin,Atorvastatin, isosorbide mononitrate, omeprazole, lactulose and paracetamol
**TP-HSV035**	53	Male	White British	Coronary artery disease	Heart attack, kidney failure.	No	Aspirin,Atorvastatin, isosorbide dinitrate, Budesonide, Clopidogrel, Lansoprazole, Losartan, metoprolol, and venlafaxine
**TP-HSV037**	65	Male	White British	Coronary artery disease	Hypertension and hypercholesterolaemia	No	Amlodipine, atorvastatin, bisoprolol, ISMN and losartan.
**TP-HSV038**	63	Male	White British	Coronary artery disease	T2DM, Heart attack, Recent infection and Ostheoarthritis	Yes	Lansoprazole, bisorolol, amlodipine, candesartan, metformin, rosuvastatin, liraglutide, empagliflozin, ranolazine, aspirin. ISMN, glyceral trinitrate and paracetamol.
**TP-HSV039**	47	Male	White British	Coronary artery disease	Heart attack, recent infection-MSA.	No	Aspirin,Atorvastatin, Bisoprolol, Omeprazole, Ramipril, isosorbide mononitrate and paracetamol

**TP-HSV040**	65	Male	White British	Coronary artery disease	Aortic valve stenosis, Asthma	No	Bisoprolol, Enalapril, Simvastatin, Allupurinol, Nifedipine and Aspirin.
**TP-HSV041**	63	Male	White British	Coronary artery disease	T2DM, heart attack, total hip replacement	Yes	Aspirin, monomil, bisoprolol, rosuvastatin and clopidogrel
**TP-HSV042**	69	Male	White British	Coronary artery disease	Unstable angina, blood cancer, larynthitis, steoarthritis, hypothyroidism, hypertension, polythemia	No	Aspirin, cinnarizine, levothyroxine, solifenacin, ISMN, bisoprolol and paracetamol
**TP-HSV043**	69	Male	White British	Coronary artery disease	Borderline T2DM, eye peripheral vision loss due to embolus, COPD, Hypercholesteroleamia	Yes	Losartan, digoxin, bisoprolol, simvastatin, elantin and Inipta
**TP-HSV044**	56	Male	White British	Coronary artery disease	T2DM, Heart attack, Asthma, left ventricular hypertrophy and acute coronary syndrome	Yes	Aspirin, allupurinol, bisoprolol, atorvastatin, doxozosin, indaprimed and ramipril
**TP-HSV045**	67	Male	White British	Coronary artery disease	Hypercholesterolaemia	No	Aspirin, atorvastatin, ramipril, tramadol, paracetamol, and oxycodone.
**TP-HSV046**	72	Female	White British	Coronary artery disease	Steroid-induced T2DM, Pancreatis, chronic anaemia, mysthenia gravis, diventiculitis	Yes	Bisoprolol, Ezetimibe, Fenofexadine, Folic acid, Isosorbide mononitrate, Lansoprazole, Linagliptin,

							Losartan, Wicoradil, Prednisolone, Methotrexerate
**TP-HSV047**	73	Male	White British	Coronary artery disease	Hypertension, Hypercholesterolaemia	No	Aspirin, Atorvastatin, Ferrous fumerate, ISMN, Ramipril, GTN Spray, Clipidogrl, Bisoprolol, and Nirondil.
**TP-HSV048**	73	Male	White	Aortic valve stenosis and coronary heart disease	T2DM, Heart attack, Thrombocytopenia, Hypertension, and Gilbert's disease	Yes	Aspirin, Atovastatin, Doxazosin, Lisinopril, Metformin
**TP-HSV049**	73	Female	White	Coronary artery disease	Kidney failure (CKD Stage 3A), Hypercholesterolaemia	No	Aspirin, Amlodipine, Ezetimibe, Atorvastatin, cansoprazole, isorsobide mononitrate
**TP-HSV050**	68	Male	White	Coronary artery disease	Osteoarthritis, appendectomy, resectomy, tonsilectomy + aderoids and ex-smoker	No	Aspirin, Bisoprolol, Rosuvastatin, Lansoprazole, Citalopram, ISMN
**TP-HSV051**	76	Male	White	Coronary artery disease	Heart attack, Hypercholesterolaemia	No	Alfuzosin, Atorvastatin, and Oxybutynin
**TP-HSV052**	75	Male	White British	Coronary artery disease	T2DM	Yes	Citalopram, Metformin, Nicorandil, Pravastatin, Aspirin, ISMN, Lansoprazole, Ramdazine, Nicorandil,

							Azithromycin, Salbutamol and Fostail
**TP-HSV053**	69	Female	White British	Coronary artery disease	T2DM	Yes	Lansoprazole
**TP-HSV054**	57	Male	Asian	Coronary artery disease	Heart attack and hypercholesterolemia	No	Aspiri, Atorvastatin, Bisoprolol, Lansoprazole, Ramipril, Ranolazone, Codeine, Paracetamol, Resomni, and Mitazapine
**TP-HSV055**	73	Male	White British	Coronary artery disease	Heart attack	No	Tamsulosin, Salmeterol, Momentazone, Bulometxre
